# Finite Element Models of Osteocytes and Their Load-Induced Activation

**DOI:** 10.1007/s11914-022-00728-9

**Published:** 2022-03-17

**Authors:** Theodoor H. Smit

**Affiliations:** 1 Department of Medical Biology, Amsterdam University Medical Centres, University of Amsterdam, Amsterdam, The Netherlands; 2 Department of Orthopaedic Surgery, Amsterdam Movement Sciences Research Institute, Amsterdam, The Netherlands

**Keywords:** Osteocyte, Finite elements, Bone fluid flow, Strain rate, Lacuno-canalicular network, Mechanotransduction, Micro-crack

## Abstract

**Purpose of Review:**

Osteocytes are the conductors of bone adaptation and remodelling. Buried inside the calcified matrix, they sense mechanical cues and signal osteoclasts in case of low activity, and osteoblasts when stresses are high. How do osteocytes detect mechanical stress? What physical signal do they perceive? Finite element analysis is a useful tool to address these questions as it allows calculating stresses, strains and fluid flow where they cannot be measured. The purpose of this review is to evaluate the capabilities and challenges of finite element models of bone, in particular the osteocytes and load-induced activation mechanisms.

**Recent Findings:**

High-resolution imaging and increased computational power allow ever more detailed modelling of osteocytes, either in isolation or embedded within the mineralised matrix. Over the years, homogeneous models of bone and osteocytes got replaced by heterogeneous and microstructural models, including, e.g. the lacuno-canalicular network and the cytoskeleton.

**Summary:**

The lacuno-canalicular network induces strain amplifications and the osteocyte protrusions seem to be stimulated much more than the cell body, both by strain and fluid flow. More realistic cell geometries, like minute constrictions of the canaliculi, increase this effect. Microstructural osteocyte models describe the transduction of external stimuli to the nucleus. Supracellular multiscale models (e.g. of a tunnelling osteon) allow to study differential loading of osteocytes and to distinguish between strain and fluid flow as the pivotal stimulatory cue. In the future, the finite element models may be enhanced by including chemical transport and intercellular communication between osteocytes, osteoclasts and osteoblasts.

## Introduction—or a Short History of Bone Physiology

### A Living Tissue

Bone has long been considered a lifeless tissue. On the outside it is compact, while on the inside, near the joints, it has an open porous structure of struts and plates. In the middle, long bones essentially are thick-walled cylinders. Leeuwenhoeck saw that compacta is ‘… *made up of very small straight and transparent pipes*’ aligned along the bone axis.[1] Havers described *longitudinal and transverse pores* and thought they were filled with *medullary oils* [2]. Albinus [3] showed that these pores contain blood vessels and called them *Haversian canals* [4]. With better microscopes, concentric lamellae were found around the canals, embedded with small cavities (*lacunae*) and minute canals (*canaliculi*) [5, 6]. Deutsch called the cavities *bone corpuscles* [5], but Virchow discovered they had nuclei and thus actually were bone cells (*osteocytes*) [6]. This implies that bone is a living tissue consisting of a mineralised matrix, embedded with blood vessels and cells.

### Mechanical Adaptation for Optimal Function

Bone and mechanical loading are strongly related [7]. Galilei already noticed that bones grow more stout as they increase in size to maintain equal stress [8]. Bourgery recognised a mechanically functional architecture in the femur and Von Meyer and Culmann pointed out that trabeculae align to principal stresses, which implies that bone bears loads with minimal weight [9]. Wolff postulated that bones can adapt to new loading conditions [10], an idea that resonated with Roux’ concept of *functional adaptation* (‘*use it or lose it*’) [11]. Frost [12] linked mechanical strain to cellular activity: when bone is deformed more than 1500 microstrain (0.15%), osteoblasts are activated to make more bone, while under 300 microstrain (0.03%) basic multicellular units (BMUs) of osteoclasts and osteoblasts are incited to resorb bone. This *Mechanostat* concept implies that bone is able to sense deformation and induce resorption and formation locally. Cowin suggested that osteocytes, spread throughout the bone matrix and highly interconnected, are in an optimal position to sense strain and signal osteoclasts and osteoblasts at the bone surface [13]. Osteocytes could be stimulated by interstitial fluid that flows dynamically between the cells and the matrix upon physiological loading [14]. The presence of such flow was already established by tracer studies [15] and the measurement of streaming potentials [16].

### Mechanosensing by Osteocytes

The isolation of osteocytes from chick calvariae around 1990 allowed studying mechanosensing in vitro [17]. Cultured osteocytes seemed a valid model, since they quickly adopt the characteristic morphology with dendritic processes and form a network on the culture dish [17]. Osteocytes appeared very sensitive to pulsatile fluid flow [18], much more than to hydrostatic pressure [19] or mechanical strain [20]. It was also found that spherical osteocytes are orders of magnitude more sensitive to mechanical strain than osteocytes flattened out on a substrate [21]. In other words, osteocytes that normally live in ellipsoid lacunae within the bone matrix [22, 23] are presumably responsive to much smaller strains than previously thought. Another interesting observation is that osteocytes are very sensitive to higher-frequency vibrations [24, 25], which suggests a mechanism in which the nucleus oscillates within the cytoplasm [26, 27]. Others report that the processes are much more mechanosensitive than the body of the osteocyte [28, 29]. As yet, there is no consensus on the precise mechanism by which osteocytes perceive and process mechanical signals.

### Signalling by Osteocytes

If osteocytes are the mechano-sensors of bone, osteoclasts are the wreckers and osteoblasts the builders. Insufficiently loaded osteocytes go into apoptosis, evidenced by a strong expression of caspase-3 [30]. Osteocytes affected by micro-cracks disconnect from the network [31], lack fluid flow that transports nutrients and waste products, and also become apoptotic [32]. They express receptor activator of nuclear factor kappa B ligand (RANKL), the principle regulator of osteoclast differentiation and activity [33]. Another relevant molecule expressed by unloaded osteocytes is sclerostin, a glycoprotein encoded by the osteocyte-specific SOST gene [34]. Sclerostin is a negative regulator of osteoblast differentiation and stimulates osteoclastic activity [35, 36]. Osteoclasts resorb damaged or unloaded matrix until they encounter mechanically stimulated osteocytes. These express nitric oxide (NO) [37, 38], which induces the retraction of osteoclasts from the bone surface [39, 40]. Other relevant molecules include cyclic oxygenase 2 (COX-2) [41], prostaglandins (e.g. PGE2) [42] and insulin-like growth factor 1 (IGF1) [43], which all play a role in the recruitment of osteoblasts. Stimulation of osteocytes downregulates the expression of sclerostin and thereby upregulates osteoblast activity [44]. Overall, the notion that osteocytes are the mechanosensors of bone and the conductors of osteoclasts and osteoblasts is well established. *How* osteocytes are mechanically triggered, however, remains an open question.

### Finite Element Modelling

Over the last few decades, computational modelling has evolved into a mature and indispensable tool of science that describes complex systems in mathematical equations. Such systems may be complex in terms of hierarchy, geometry or material properties, and most biological systems combine them all. Computational modelling allows investigating system behavior under regular and extreme conditions, extrapolating theoretical assumptions, and formulating new hypotheses. This is particularly helpful in non-linear and multi-hierarchical systems, where experimental observations are difficult or impossible to obtain. For bone, with the mechanosensitive osteocytes buried deep inside the mineralised matrix, computational modelling allows calculating local stresses, molecular signalling and the transport of nutrients and waste products.

In finite element modelling, complex systems are divided into a large (finite) number of small elements that are mutually connected at their nodes and element boundaries. The values of quantities like stress, temperature, electric current or chemical concentration, are described at the nodes and boundaries of each single element. Provided that the behaviour of each element can be calculated adequately, the behaviour of the entire system is rendered by the summation of them all. Reversely, the system affects the behaviour of each single element, because each node belongs to several elements. Good introductions of Finite Element modelling in biology are [45–47].

### Aim of the Review

This review addresses the capabilities and challenges of finite element models of osteocytes and their load-induced activation, at different hierarchical levels. The first part addresses homogenized, supra-cellular models of bone adaptation in relation to mechanical loading. Then finite element models of isolated, single osteocytes under various loading conditions are discussed. Finally, we consider osteocytes in situ, including the canaliculi that enclose the osteocyte protrusions and allow interstitial fluid flow. A glossary of engineering terms is provided in Table 1.

**Table 1:** Glossary of engineering terms

**Physical quantity**	**Meaning**	**Unit**
Force	Mechanical push or pull on an object	N
Stress	Force per unit area	N/mm^2^, MPa
Normal stress	Stress perpendicular to object surface	N/mm^2^, MPa
Shear stress	Stress parallel to object surface	N/mm^2^, MPa
Hydrostatic pressure	All-sided pressure (like object under water)	N/mm^2^, MPa
Principal stress	Normal stress in direction at which the shear stress is zero (i.e. pure compression or tension)	N/mm^2^, MPa
Strain	Deformation	(% or °)
Normal strain	Elongation or shortening of object, divided by original length (Δl/l)	(%)
Microstrain	Strain Δl/l = 0,000001 (1*10^−6^) 1% elongation = 10,000 microstrain	(%)
Shear strain	Angular distortion of an object caused by a shear stress (e.g. fluid flow)	° (angle)
Strain rate	Changes of strain in time (as in dynamic loading)	%/s
Elasticity	Ability of an object to resume its shape after releasing a force applied to it	
Elastic (Young’s) modulus	Resistance of a material against deformation (material stiffness)	N/mm^2^, MPa
Visco-elasticity	Property of materials that have both viscous (dissipative) and elastic characteristics under mechanical stress	
Poro-elasticity	Property of porous solids in which fluids flow under mechanical stress. All biological tissues, including bone, are poro-elastic	
Porosity	Fraction of voids within a solid body	%
Permeability	Ability of fluid to transmit fluids (inverse of resistance against fluid flow)	mm^4^ / N s
Streaming potential	Electrical potential that occurs when charged (ionized) fluids flow through a tissue	

## Homogenized Models of Mechanical Bone Adaptation

Finite element analysis (FEA) was introduced in orthopaedic biomechanics in 1972 [48] to assess stresses in bones and (artificial) joints [49]. Bone adapts its density and structure in response to changed mechanical cues, and FEA is the perfect tool to study that. Stresses, strains and their derivatives can feed back into the model to optimise the geometry and density of the matrix. A practical application is the optimization of implant design, using a window of stress as objective function [50, 51]. Finite element optimization algorithms also allow studying the fundamentals of the mechanical adaptation of bone [52]. Are bones indeed optimised structures and if yes: optimised to what? For this question, it is useful to distinguish between trabecular and cortical bone models, because of substantial differences in density and geometry.

### Trabecular Bone Remodelling

The earliest finite element analyses modelled trabecular bone as a homogeneous tissue with apparent density ρ, defined as the ratio of bone volume to total volume in a volume of interest [53]. For example, E (elastic modulus E can be calculated as E = .3790ρ^3^) [54]. Stresses in all elements are calculated; the density of each element is adapted to equalize deformation energy; and stresses are recalculated until the deformation energy is the same in the entire bone. This way, apparent bone density and orientation were predicted commensurate with the real trabecular bone structure in a femur [55], which suggests that bone is indeed a mechanically optimised tissue.

The alignment of a single trabecula to mechanical loading is easy to understand: when loaded off-axis, a strut is bent and experiences stress concentrations on its concave sides, and decreased stresses on its convex sides. Following the principle of functional adaptation, stress concentrations induce bone formation, and decreased stresses lead to resorption. This process continues until stresses and strains are the same over the entire trabecula, which is when it is aligned to the applied force [56]. With the availability of micro-CT scanners and large parallel computer systems, this principle can be applied to very large micro-FE models that represent whole human vertebrae or femora in detail [57, 58].

A similar relation between strain and cellular activity exists in *remodelling*, during which damaged bone is replaced in a sequence of resorption and deposition. When microcracks are removed by osteoclasts, elevated strain levels are found at the bottom of the lacuna, which increase as the lacuna gets deeper. By contrast, decreased stress levels appear in the longitudinal direction of the trabecula. This drives osteoclasts to continue their activity in the loading direction, followed by osteoblasts that deposit new bone. This explains the alignment of lamellae in trabeculae [59].

### Cortical Bone Remodelling, or the Formation of Osteons

**Fig. 1:**
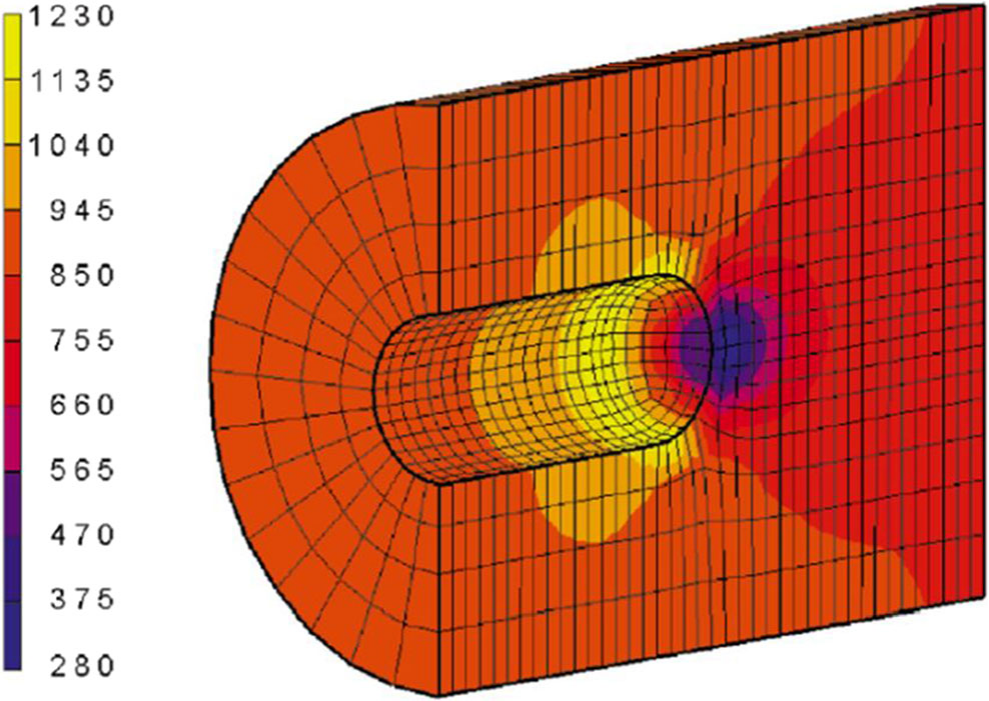
Equivalent strain around the tip of a tunnelling osteon. Decreased strain is indicated by blue and correlates with resorption, high strain is yellow and correlates with bone deposition [59]

When cortical bone is overloaded and microcracks are formed [60], a similar repair process occurs. Here, remodelling proceeds by tunnelling, where osteoclasts excavate a volume of damaged bone to allow the deposition of new bone. The result is a *Haversian system* or *osteon* [61]. Similar to trabeculae, osteons align in the direction of loading [62, 63], which can be understood by looking at the stress concentrations around the tip of the osteon (Fig. 1): in front of the tip mechanical strains are substantially decreased, while on the lateral edges stress concentrations occur that shifts bone resorption to bone formation.

Other models of cortical bone remodelling were developed that confirm the role of mechanical loading in osteon formation. For example, it relates the inner- and outer diameter of osteons to the magnitude of the local stress, with smaller osteons and tinier tunnels for higher stress [64], and larger osteons and higher porosity at reduced loading [65, 66]. Overall, the algorithms present remodelling as a process of local self-organisation, rather than global optimization. Yet, the result is a minimum-weight structure, because eventually all parts of the tissue become equally stressed.

### Poro-Elastic Models of Interstitial Fluid Flow

The mineralised bone matrix not only contains a network of osteocytes, but also free, interstitial fluid that flows upon mechanical loading of the bones [14]. Weinbaum suggested that this fluid flow stimulates osteocytes [67]. To consider the role of interstitial fluid flow in osteonal remodelling, homogenized poro-elastic models were developed that can visualise the fluid flow patterns within the bone tissue and relate them to the activity of osteoclasts and osteoblasts [68]. Fluid flow around the tip of the excavating osteon appears strongly related to deformation energy: low in front of the osteon in the direction of loading and high perpendicular to that [69]. Further, fluid flow is high at the inner wall of the osteon, but almost zero at the cement line [70, 71]. This puts a limit on the transport to and from the osteocytes and (thus) their role in bone remodelling.

For the closing osteon, the picture is somewhat different. Bone apposition decreases as filling of the osteon proceeds [72, 73], but the fluid flow under mechanical loading remains constant [74]. This suggests that fluid flow is unrelated to osteoblast activity. By contrast, shear strain rate declines linearly with bone apposition, which indicates that deformation of the osteocytes is the more relevant mechanical cue driving bone adaptation.

Homogenized, supra-cellular finite element models of bone allow mechanobiological studies at the level of trabeculae and osteons, more particular the differential activation of osteoclasts and osteoblasts. While there is general consensus that osteocytes function as mechanosensors and conduct the activity of osteoclasts and osteoblasts, the finite element models used lack detail, e.g. the osteocytes and the type of loading that turns them on. Another limitation of the current models is that they only include mechanical cues and disregard a possible role and transport of molecular cues, like RANKL, SOST, NO, COX2, IGF1 and others factors which are strongly related to the mechanical stimulation of osteocytes. Also, the removal of cellular waste products appears to be a factor if importance. Such transport of molecular agents can be modelled by Cellular Potts models, which govern the diffusion and convection of signals to and from cells [75]. Finally, it would be interesting to explicitly model the deposition of unmineralized collagen (osteoid) and the effect of lower tissue stiffness on the activity of osteoblasts in a negative feed-back loop.

## Finite Element Models of Single Cells

To understand mechanosensing, one should monitor the deformation of an isolated cell subjected to a well-defined mechanical load, create a computer model and tune the parameters to optimally fit the observations. Many techniques have been developed to hold and load a cell, including micropipette aspiration, optical tweezers, cyto-indenters, fluid shear stress, vibrations and strain (for reviews see [76–78]). Also, various single cell computer models have been developed, roughly divided in continuum models that describe the cell as a homogeneous mass, and microstructural models that explicitly consider the cytoskeleton, the nucleus, the membrane and other organelles (reviewed in [76, 79]). While many types of cells have been probed and modelled [77], this review focuses on osteocytes and an osteocyte-like cell-line (MLOY4; [80]).

### Continuum Models of the Osteocyte

For osteocytes, a convenient setting may be a single cell on a flat surface, which can be subjected to tension, fluid flow, micropipette aspiration, vibrations and other loading conditions [18, 21, 27, 81–83]. Osteocytes have been modelled as a linear elastic material [84], but essentially show time-dependent behavior [85, 86]. Qiu modelled an MLO-Y4 osteocyte as a homogeneous, viscoelastic solid characterized by three parameters: an elastic modulus to describe immediate deformation; an equilibrium modulus to describe the long-term response; and viscosity to quantify relaxation rate [87]. Using the actual cell geometry instead of an assumed idealised shape, cell behavior under fluid flow could accurately be described [88]. Nguyen and Gu used modified Standard neo-Hookean Solids to describe osteocytes subjected to dynamic indentation [89]. Hyperelastic elements were used to account for large deformations and cells were considered compressible, assumptions that are relevant for low strain rates [89]. Thus, different non-linear models can be used to accurately describe osteocyte behavior under dynamic loading. This is commensurate with studies that use visco-hyperelastic, poro-hyperelastic or even poro-visco-hyperelastic descriptions of cell behavior [90, 91]. Sophisticated continuum models are useful to accurately quantify the stiffness of healthy and diseased cells [92] However, they fall short in describing mechanosensing.

### A Microstructural Model

**Fig. 2:**
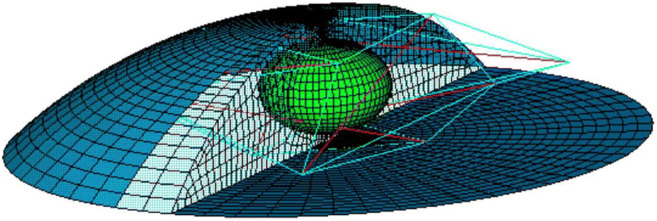
3D finite element model of an adherent cell [99]. The model includes a nucleus (green), micro-filaments (blue lines), micro-tubules (red lines), cytoplasm (transparent) and membrane (dark blue)

How cells sense mechanical stress and transform it into biochemical signals essentially depends on how they resist deformation. Eventually, a change of shape must be communicated to the nucleus in order to provoke a molecular response. This is best accomplished by the cytoskeleton, which connects the integrins, cadherins and primary cilium in the cell membrane to the nucleus [93]. Indeed, it is well established that disrupting the cytoskeleton eliminates mechanosensing in osteocytes [94]. One of the earliest models explicitly considering the cytoskeleton was presented by McGarry [95]. It includes a *tensegrity* network [96] of six compression struts and 24 tension elements representing the microtubules and microfilaments, respectively (Fig. 2). The cell shows increasing stiffness (*strain hardening*), commensurate with experimental observations [97]. It appeared that 0.6 Pa fluid shear stress results in eight times higher strains at the apical surface of the cell than 1000 μstrain (0.1%) substrate tension, thereby confirming experimental observations that fluid flow is more stimulatory to an osteocyte on a flat surface than substrate strain [82, 98, 99].

### Active Intracellular Stress

Reynolds modelled micropipette aspiration of an osteocyte adhered to a flat surface [100]. This loading condition is obviously non-physiological and involves extreme deformations (up to 100%) of both cell and nucleus. Nevertheless, cell behavior was accurately described, provided that contractile actin stress fibres were included into the model; a passive viscoelastic model was incapable of predicting aspiration length. Reynolds also pointed out that the cell nucleus is highly deformable, a conclusion substantiated for more physiological loading conditions in a recent study on chondrocytes [101]. Intracellular stress was also considered in a study on the differentiation of osteoblasts to osteocytes [102], cells with a close biological relationship but highly different morphology. Mullen reported that the area of focal adhesions correlates with intracellular stress, and both are more prominent on stiffer substrates. There were more focal adhesions on spread cells than on dendritic cells, but spread cells have focal adhesions close to the nucleus, while the dendritic cells have them in the processes. Further, cells cultured on a soft substrate actively adapt a dendritic structure in order to achieve higher internal stress. This implies that active stress fibres are indeed critical elements to describe the cellular response to mechanical cues [103].

### Vibration Studies

Osteocytes are highly responsive to vibrations. Rubin and Lanyon reported that bone is more responsive to dynamic than to static loading [104, 105], and trabeculae in sheep long bones get substantially denser after exposure to low-magnitude high-frequency vibrations [106]. Bacabac observed high sensitivity of osteocytes to vibrations in vitro and proposed that the nucleus vibrates within the cytoplasm to excite the mechanotransduction machinery of the cell [26]. Uzer simulated this in a finite element model via dynamic stress analysis [27]. The osteocyte on a flat surface included a nucleus with a stiffness four times that of the cytoplasm [107]. As vibrations had a frequency of at least 30 Hz, the cell membrane, cytoplasm and nucleus were all modelled as linear elastic materials. Vibrations induced acceleration-dependent displacements of the nucleus within the cytoplasm, while the effect of fluid shear stress was minimal. This essentially confirms the suggestion by Bacabac [26], but there were also limitations of the model, such as a lack of a cytoskeleton and the idealised geometries of cell and nucleus. Wu constructed a 3D cell model from confocal serial images of a MLO-Y4 cell and explicitly included F-actin [83]. Shape changes in cell and nucleus were marginal, but the displacement of the nucleus within the cytoplasm was not assessed. Instead, it was found experimentally that the F-actin at the nucleus periphery became dispersed at high frequencies (90 Hz), which fits with the notion that osteocytes are less responsive at this frequency. It also supports the idea that an intact cytoskeleton and a direct connection to the nucleus are mandatory for mechanosensing [108].

Single osteocyte finite element models are sophisticated but have various limitations, some of which are addressed in the next section (including their three-dimensional shape and the more realistic tissue environment and loading conditions). The models are currently focused on simulating cellular deformation upon mechanical loading, but the more relevant question may be how mechanical cues are actually transduced to the nucleus and induce specific signalling pathways. This may allow discriminating between the various mechanotransduction mechanisms proposed in literature. As for the three-dimensional shape of the osteocytes, it seems necessary to include more details of the cytoskeleton, in particular the development of actin fibres in the protrusions and the deformation of the nucleus itself. Modelling the differentiation of an osteoblast to an osteocyte, during deposition of osteoid, may also provide valuable insights into the function of the cytoskeleton and the osmotic pressure of the cytoplasm.

## In Situ Models

Sophisticated finite element models of single, isolated osteocytes have been developed to describe their deformation under various loading conditions and study mechanisms of mechanosensing. Cellular deformations are well described by assuming viscoelastic material properties [88, 89], but it requires explicit modelling of the nucleus, cytoplasm, cytoskeleton and membrane to study mechanosensing [99, 100, 102]. Further, the conditions modelled are rather unphysiological and strongly deviate from the situation in vivo. Osteocytes in bone not only have a different shape (three-dimensional with many dendrites), but are also surrounded by fluid and tightly embedded in a pericellular and a mineralised matrix. Osteocytes thus experience entirely different loading conditions than the isolated cells in vitro. Therefore, three-dimensional in situ models of osteocytes are required with protrusions in a mineralised matrix.

### Strain Amplification

A central paradox in bone physiology is that osteocytes are considered the conductors of adaptation and repair, but seem insensitive to strains caused by the activities of daily life [98]. Nicolella [109] pointed out that strains have been measured in vivo under the assumption that bone is a homogeneous material [110]; this is challenged, however, by the microstructural organisation of Haversian canals and the lacuna-canalicular network [111]. Nicolella measured the deformations of the matrix around osteocyte lacunae and found that local strains can be an order of magnitude larger than the global, average strains [109]. This was further substantiated in an idealised, three-dimensional finite element model of a single osteocyte with canaliculi running through the matrix [112]. Strain in the matrix was amplified by more than three times and more so for larger inhomogeneities. The soft perilacunar matrix attenuated this effect. The strain at the base of the canaliculi could be as high as 1.0% (10,000 μstrain), well in the range that osteocytes are able to detect [98]. This supports the idea that the protrusions, rather than the cell body, could be the site of mechanosensing [28, 29]. Wang considered dynamic loading conditions in a comparable finite element model and found that the strain amplification also increased with load and frequency [113].

Verbruggen built realistic finite element models of osteocytes based on confocal imaging [114]. Using a resolution of 0.125 μm in the x-y plane and 0.410 μm in the z-direction, cells were modelled with 6–10 irregularly shaped protrusions within an unmineralized pericellular matrix and a mineralised extracellular matrix. The strains around these osteocytes were 350–400% larger than those around idealised osteocyte models. Furthermore, a substantial part of the osteocyte experienced a strain of more than 3500 μstrain, which suggests that also the cell body can be mechanosensitive. The high-resolution confocal images revealed constrictions along the canaliculi, which amplified strains of the protrusions by an additional 50–420% [114]. Varga used synchroton X-ray phase nano-tomography to reconstruct the lacuno-canalicular network of cortical bone at a resolution of 50 nm [115]. They found a high number of evenly distributed canaliculi sprouting from each lacuna (89 ± 25) and also regular constrictions of the canaliculi. The strain concentrations in this model were also substantial, up to factor 70, which surely should lead to microfractures [116] and remodelling [60, 117]. Kola showed that the orientation and the size of the lacunae also affect strain amplification, with higher strains for larger and less well aligned osteocytes [118].

### Fluid Flow

**Fig. 3:**
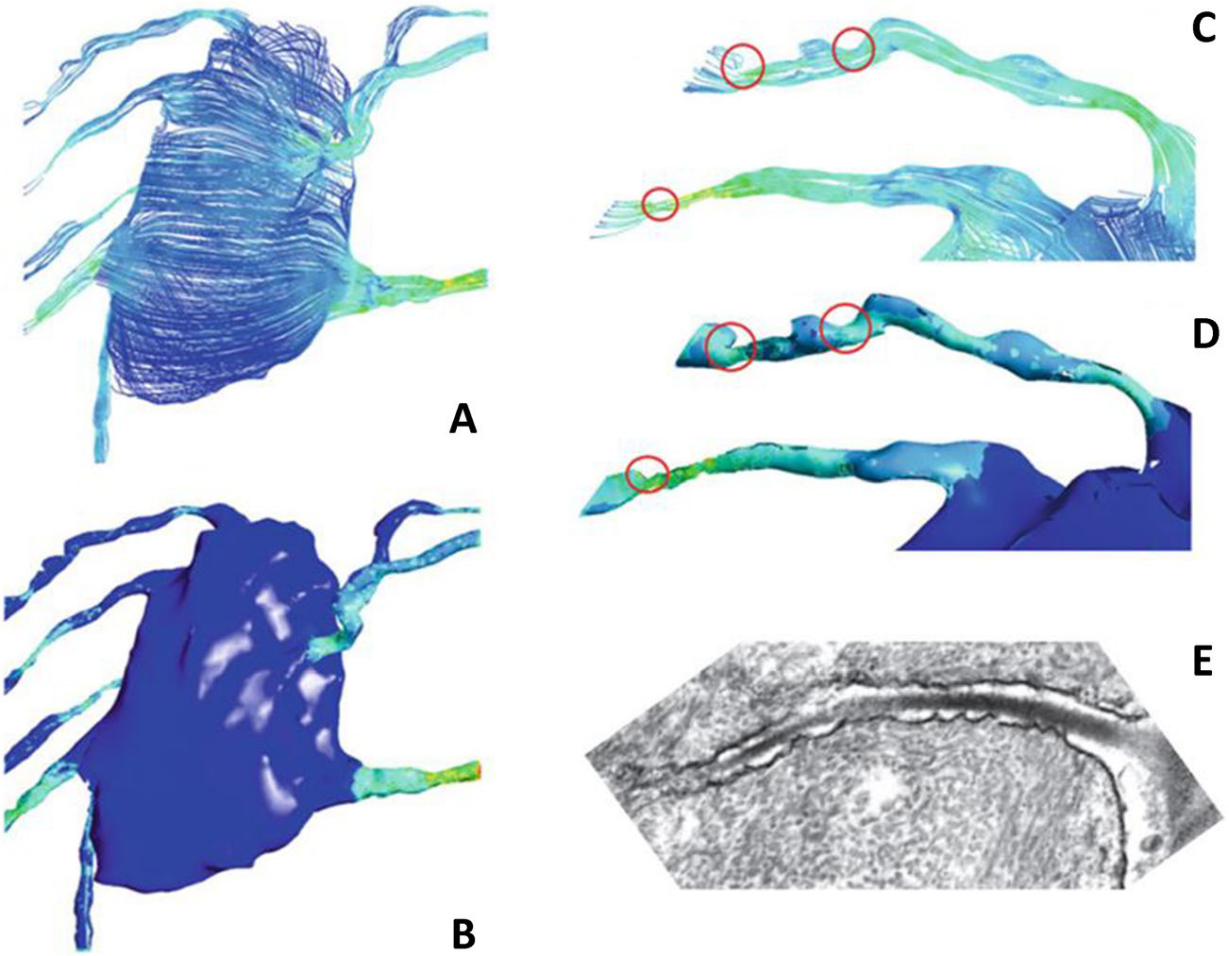
Osteocyte stimulation by fluid flow [123]. **A** Fluid flow around a single osteocyte. **B** Resulting shear stress. **C** Zoom of fluid flow around protrusion showing restrictions in canaliculi that work as stress concentrations. **D** Increased shear stress. **E** Electron microscopy of a single canaliculus containing an osteocyte protrusion. Note the irregular boundaries [128]

The lacuno-canalicular network contains osteocytes, unmineralized pericellular matrix and interstitial fluid that transports nutrients and waste products [14–16]. Computational modelling of this fluid flow, however, requires techniques that go beyond Biot’s theory of poro-elasticity [119]. Early models of fluid flow around osteocytes are two-dimensional [120], but the lacuno-canalicular network is essentially three-dimensional [121]. Anderson modelled a single osteocyte with few canaliculi and no consideration of the pericellular matrix [122]. As Reynolds numbers are very low at submicron dimensions, Navier-Stokes equations were used to calculate fluid flow and shear stress along an idealised, ellipsoid osteocyte and straight protrusions. It was observed that the cell body in the lacuna experienced virtually no shear stress, but merely a hydrodynamic pressure. The highest stresses were found where the processes sprout from the cell body. Although the model was highly simplified, the conclusions were confirmed by more realistic models [123–126]. Verbruggen [123] explicitly studied the interaction between the solid and fluid phases in bone, allowing to not only calculate fluid velocity and wall shear stress, but also the strains in the cell body and protrusions (Fig. 3). The more realistic geometry of the lacuno-canalicular network resulted in higher shear stresses and hydrodynamic pressure. Joukar [124] observed that shear strains also depend on osteocyte morphology and the direction of loading. Vaughan [127] investigated the stimulation of primary cilia in osteocytes. A cilium extending from the cell body can function as a mechanosensor under fluid flow in vitro [93], but the lacuno-canalicular network presents an entirely different situation, with virtually no fluid flow around the cell body. However, a cilium that connects to the wall of the lacuna can still be highly stimulated and thus be functional [127].

With finite element models of osteocytes and their environment becoming more detailed and more realistic, computational costs are increasing, both in time and financially. Still, the number of protrusions in the model of figure 3 is only eight, while estimations for the number of protrusions of real osteocytes are at least an order of magnitude higher [115]. One may wonder, however, whether more detailed models are required to better understand osteocyte physiology, or whether simpler models may elucidate the main principles of mechanotransduction. The downside of such an approach may be that the models and their computed results may become trivial, e.g. that tinier canaliculi result in higher shear stresses.

## Osteocyte Processes and Canaliculi

Strain induced fluid flow produces the highest shear stress in the canaliculi, which are the smallest pores with the highest geometric inaccuracy. The earliest models assume a straight, cylindrical tunnel filled with a central cell process and fluid flow [120, 122], but high-resolution scanning studies showed that canaliculi are curved, contain an unmineralized pericellular matrix and have ultrastructural irregularities that affect fluid flow and shear stress along the protrusions (Fig. 3E) [121, 128, 129].

Anderson and Knothe-Tate built finite-element models of single canaliculi based on high-resolution transmission electron microscopy and found that the irregularities induced stress peaks about five times those in an idealised model [130]. They also found that the width of the canaliculi and the diameter of the protrusions vary substantially. You [128] had observed that irregularities could extend all the way to the protrusions and in fact physically connect. Based on this, Wang created a model that predicts that strains caused by the sliding of actin filaments along the attachment points are about two orders of magnitude larger than the strains of the whole tissue [131]. Kamioka used ultra-high voltage electron microscopy to visualise the canaliculi and found highly irregular channels, which had profound effects on fluid flow and shear stress [129]. However, they did not observe attachments of the cell protrusion to the canalicular wall, and thereby seem to refute Wang’s theory of direct strain amplification. Nevertheless, multiple studies confirmed that the canaliculi contain physiological irregularities that strongly increase fluid flow shear stress along the protrusions, an observation that could solve the bone mechanosensing paradox.

## Multiscale Models of Cortical Bone

The steady increase of computing power allows for more detailed models with more finite elements. Bone is a hierarchical tissue, where loading of whole bones results in fluid flow and cellular deformations at the submicron scale. Cortical bone has two levels of porosity, with Haversian canals about 50 μm wide and canaliculi in the order of 200 nm [132], which affect the physics and biochemistry of mechanotransduction. Early multiscale models of cortical bone are rather schematic [133, 134], but conclude that the micropores are the stress enhancers. Vaughan created a multiscale model of an osteon and observed that the inhomogeneous structure leads to vastly different stimuli at the different levels, depending on their exact location [135]. For example, osteocytes in the vicinity of micropores sense strains about nine times the applied global strain. They conclude that osteocytes within the bone matrix receive vastly different cues and that only a subset of osteocytes (those near the porosities) can function as mechanosensor. Recent multiscale finite-element studies included interstitial fluid flow and reached similar conclusions: fluid flow is highest close to the Haversian canals, and close to zero at the cement line [125, 136, 137]. Thus, the fluid shear stress that an osteocyte perceives highly depends on its position, a conclusion that could also be drawn from homogenized Biot-type poro-elasticity models [69, 74].

## Microdamage and the Interruption of osteocyte Connectivity

While microporosities affect strain fields and fluid flow around cells, microcracks have an even larger effect on osteocytes. Bone remodelling is strongly related to microdamage [60, 138], because it disrupts the intercellular connections between osteocytes and the fluid flow through the lacuno-canalicular network [60, 117]. Prendergast and Huiskes used a two-dimensional finite element model to show that microdamage indeed elevates the deformation of lacunae [31]. Donaldson built microstructural finite element models based on micro-CT imaging, where high-resolution image voxels are directly converted into hexahedral finite elements [139]. Microdamage was implemented by deleting elements that were stressed beyond a certain threshold. Crack propagated from lacuna to lacuna, and stress was relieved in the adjacent tissue once the microcrack was formed. Although no osteocytes were modelled, microdamage clearly affects the local strain fields.

While local stressing of osteocytes may be enhanced by microdamage, the major effect may be that the transport of nutrients, waste products and signalling is disturbed. Ridha modelled osteocyte signalling and its inhibition by microcracks and found that such a mechanism could explain the activation of osteoclasts and osteoblasts on the bone surface [140]. Reduction in the number and connectivity of canaliculi affects diffusion and convection of fluid flow, nutrients and waste products. The shape of the canaliculi, however, (i.e. bending and tortuosity) did not affect pericellular fluid flow through the network [141]. Schurman explicitly modelled an osteocyte with protrusions within the lacuno-canalicular network and found that expanding the pericellular space could rescue fluid flow and the mechanosensation by osteocytes.

## Discussion

*All models are wrong*, *some are useful* [George Box]. Finite element models are useful for calculating physical cues that cannot be measured, or only with extraordinary effort. Also, it allows extrapolating processes in space and time, thereby estimating possible consequences of choices or hypotheses. In bone physiology, it is practically impossible to measure the physical cues that stimulate a single osteocyte inside the extracellular matrix and relate that to the activation of osteoclasts and osteoblasts during bone remodelling. Also, the osteocyte itself is too complex to understand how mechanical signals transduce into the nucleus and provoke a biological response. The statement that all models are wrong refers to the assumptions that underlie them and the degree to which they are (in)correct or can be validated.

Since the introduction of finite element models into orthopaedic biomechanics [48], computing power and microscopic detail have increased tremendously. Early models presented bones and implants as homogeneous masses, the most recent models are multiscale, including Haversian canals and the lacuna-canalicular network, the osteocytic syncytium within and details like actin fibres in the protrusions and the constrictions of the canaliculi [141]. Also, the process of mechanical adaptation and the physiology of mechanosensing can be studied. Geometries have become much more realistic as a result of high-resolution imaging techniques [129, 135, 142]. The material properties, on the other hand, are necessarily simplified because we do not exactly know the mechanical properties of the extracellular bone matrix, the unmineralized pericellular matrix and the living osteocytes with their numerous protrusions and active stress fibres. We assume, furthermore, that the interstitial fluid is similar to salt water, and neglect the transport of ions and complex molecules [16, 143]; we do not know if and how they are restrained by the glycocalyx between the osteocytes and the electrically charged matrix. Finally, we assume that the laws of physics (in particular Darcy’s Law of fluid flow) are applicable on the submicron scale.

Osteocyte physiology has been considered on various scales: supracellular to assess heterogeneous stresses and strains; cellular to study mechanotransduction through actin fibres and the nucleus; and submicron to address interstitial fluid-flow along the protrusions. One insight obtained is that the vascular and lacuno-canalicular porosities in the cortex induce stress concentrations that increase average whole-bone strains to levels that can be sensed by osteocytes [112]. Further, it appears that osteocytes at the inner border of osteons perceive more fluid shear stress [69, 135], while those at the cement line rather experience hydraulic pressure [120, 123]. Also, the osteocytes near microcracks or in front of a remodelling osteon are hardly stressed, while those just behind the remodelling tip experience increased stress [59]. Visualizing such supracellular strain fields is useful, because they can be related to the differential activity of osteoclasts and osteoblasts. Models of single osteocytes subjected to mechanical cues in vitro are rather unphysiological, but show that a cytoskeleton that connects integrins at the cell membrane to the nucleus is a likely and efficient way of mechanotransduction. Finally, models of fluid flow in the canaliculi show that constrictions of the matrix can induce stress concentrations, but we do not know whether they are biologically relevant. In general, one may state that finite element models are able to visualise stresses and strains as well as fluid flow within the bone matrix, but their biological relevance is as yet unclear.

Where do we go from here? Can finite element models answer our biological questions? Are osteocytes stimulated by strain, fluid flow, streaming potentials or chemical signalling? In vivo, it all happens at the same time and finite element models only seem to confirm any hypothesis. There are good arguments that osteocyte protrusions are the mechanosensors, but the cell body may be mechanosensitive as well. Rather than trying to simulate what happens to an osteocyte inside the bone matrix under mechanical loading, it may be necessary to focus on differential loading conditions, like the tunnelling osteon or the presence of microcracks. Also, with the upcoming technology of 3D bioprinting, one may create artificial conditions for osteocytes and discriminate between various stimuli, for example fluid flow vs. strain vs. streaming potentials around a geometrical heterogeneity, or varying permeability of the matrix with constant porosity. Finite element models may help to visualise and clarify their differential conditions. Furthermore, it may be relevant to include chemical transport and the activation of osteocytes, osteoclasts and osteoblasts. Cellular Potts models [144, 145] which describe intercellular communication may be a useful addition to the exclusive mechanical approach of finite element models.
